# The Role of Nibrin in Doxorubicin-Induced Apoptosis and Cell Senescence in Nijmegen Breakage Syndrome Patients Lymphocytes

**DOI:** 10.1371/journal.pone.0104964

**Published:** 2014-08-13

**Authors:** Olga Alster, Anna Bielak-Zmijewska, Grazyna Mosieniak, Maria Moreno-Villanueva, Wioleta Dudka-Ruszkowska, Aleksandra Wojtala, Monika Kusio-Kobiałka, Zbigniew Korwek, Alexander Burkle, Katarzyna Piwocka, Jan K. Siwicki, Ewa Sikora

**Affiliations:** 1 Laboratory of the Molecular Bases of Aging, Nencki Institute of Experimental Biology, Polish Academy of Sciences, Warsaw, Poland; 2 Molecular Toxicology Group, Department of Biology, University of Konstanz, Konstanz, Germany; 3 Laboratory of Cytometry, Nencki Institute of Experimental Biology, Polish Academy of Sciences, Warsaw, Poland; 4 Department of Immunology, Maria Sklodowska-Curie Memorial Cancer Center and Institute of Oncology, Warsaw, Poland; Columbia University Medical Center, United States of America

## Abstract

Nibrin plays an important role in the DNA damage response (DDR) and DNA repair. DDR is a crucial signaling pathway in apoptosis and senescence. To verify whether truncated nibrin (p70), causing Nijmegen Breakage Syndrome (NBS), is involved in DDR and cell fate upon DNA damage, we used two (S4 and S3R) spontaneously immortalized T cell lines from NBS patients, with the founding mutation and a control cell line (L5). S4 and S3R cells have the same level of p70 nibrin, however p70 from S4 cells was able to form more complexes with ATM and BRCA1. Doxorubicin-induced DDR followed by cell senescence could only be observed in L5 and S4 cells, but not in the S3R ones. Furthermore the S3R cells only underwent cell death, but not senescence after doxorubicin treatment. In contrary to doxorubicin treatment, cells from all three cell lines were able to activate the DDR pathway after being exposed to γ-radiation. Downregulation of nibrin in normal human vascular smooth muscle cells (VSMCs) did not prevent the activation of DDR and induction of senescence. Our results indicate that a substantially reduced level of nibrin or its truncated p70 form is sufficient to induce DNA-damage dependent senescence in VSMCs and S4 cells, respectively. In doxorubicin-treated S3R cells DDR activation was severely impaired, thus preventing the induction of senescence.

## Introduction

Nijmegen Breakage Syndrome (NBS) is a rare autosomal recessive disorder characterized by genomic instability and increased risk of haematopoietic malignancies observed in more than 40% of the patients by the time they are 20 years old [1]. NBS is caused by mutations in the *NBN* gene (originally designated as *NBS1*) encoding nibrin. More than 90% of the patients are homozygous for the same mutation (c.657-661del5) what results in the formation of two truncated fragments of the 95 kDa nibrin: 26 kDa N-terminal fragment (p26-nibrin) and 70 kDa C-terminal fragment (p70-nibrin), which are produced by alternative initiation of translation at a cryptic upstream start codon. This mutation is actually hypomorphic as the truncated p70-nibrin is able to retain some of the vital cellular functions of the full-length protein. The truncated p70-nibrin can form the MRN (Mre11-Rad50-Nbs1) complex with two other proteins, Mre11 and Rad50 [2], [3]. However, null mutation of the *Nbn* gene is lethal in mice [4].

Stress-induced premature senescence (SIPS) is a relatively fast, telomere erosion independent, process. Among its characteristic features we can distinguish irreversible growth arrest, altered cell morphology, DNA foci formation, activation of senescence-associated β-galactosidase (SA-β-Gal) and senescence associated secretory phenotype-SASP (reviewed in [5]). Recently, it was shown that double-strand DNA breaks (DSBs), after induction of the DNA damage response (DDR), are crucial for cellular senescence [6]. Briefly, upon DSB induction ataxia telangiectasia mutated (ATM) kinase is activated. The activated kinase phosphorylates nibrin at its Ser 343 residue and H2AX histone, at its Ser 139 residue (γH2AX). Phosphorylated nibrin forms a trimeric complex (MRN) along with Mre11 and Rad50, which is recruited to the vicinity of DSBs where nibrin interacts with γH2AX [7]. Ultimately, Chk1, Chk2 (checkpoint kinase 1 and 2, respectively) and p53 are activated. p53 promotes senescence (when DNA damage is irreparable) *via* transactivation of *CDKN1A*, which encodes the cyclin dependent kinase inhibitor p21 [5].

DDR activation, not only can lead to senescence but also to transient cell cycle arrest and DNA repair or apoptosis. Improperly functioning DDR often results in increased radiosensitivity, genomic instability and cancer development. Since NBS1 deficient cells are characterized by genomic instability and NBS patients suffer from haematopoietic malignancies, we hypothesized that the molecular pathways leading to DNA damage-induced senescence might be impaired in patients affected with this disease. Most cell lines derived from NBS patients were established following transformation with viral oncogenes, which inhibit key regulatory genes such as the tumor suppressor gene proteins p53 and pRb, thus allowing the cell to bypass the senescence program and become immortal [8]. Accordingly, spontaneously immortalized T cell lines, S3R and S4, carrying the same mutation within the *NBN* gene, but with a seemingly functional p53/p21 response after gamma irradiation [9], are a very useful cellular model in studying the mechanisms of DNA damage-induced senescence. Therefore we used two cell lines derived from NBS patients (S3R and S4) and the control, L5 cell line (spontaneously immortalized spleenocytes obtained from a healthy donor) to examine if they are prone to DNA damage-induced senescence. To induce DNA damage and DDR activation we used doxorubicin, which is a DNA damaging agent acting through different mechanisms. It can lead to the formation of direct and indirect DNA damage through: intercalation into DNA, DNA binding and alkylation, DNA cross-linking, interference with DNA unwinding or DNA strand separation, helicase activity as well as inhibition of topoisomerase II and generation of free radicals [10].

## Materials and Methods

### 1. Cell lines

The spontaneously immortalized T cell lines: S3R and S4 were established from peripheral blood mononuclear cells (PBMC) derived from NBS patients homozygous for the 657del5 mutation of the *NBN* gene [9] and the L5 cell line was established from the spleen of a healthy donor as described previously [9], [11]. All of the cell lines were cultured in the RPMI 1640 medium (Gibco, Life Technologies, Warsaw, Poland) supplemented with 10% FCS (Biochrom, Biomibo, Warsaw, Poland), 50 µg/ml gentamycin (Sigma, Poznan, Poland), 2 mM glutamine (Sigma, Poznan, Poland) and 20 U/ml of IL-2 (R&D, Biokom, Warsaw, Poland). Human vascular smooth muscle cells (VSMCs) were obtained from Lonza (Basel, Switzerland). hVSMC were grown in SmBM medium (Lonza, Basel, Switzerland). S3R, S4 and L5 cells were seeded at a density of 0,2×10^6^/ml 24 h before doxorubicin (Sigma, Warsaw, Poland) treatment. VSMCs were seeded at a density of 2×10^3^/cm^2^ 24 h before transfection.

### 2. DNA content and cell cycle analysis

For DNA analysis the cells were fixed in 70% ethanol and stained with PI solution (3,8 mM sodium citrate, 50 µg/ml RNAse A, 500 µg/ml PI in PBS). All of the used agents were purchased at Sigma Aldrich (Poznan, Poland). DNA content was assessed using flow cytometry and analyzed with the CellQuest Software. 10000 events were collected per sample (FACSCalibur, Becton Dickinson, Warsaw, Poland).

### 3. Immunoprecipitation

S3R and S4 cells were lysed with modified RIPA buffer [12]. Equal amounts of protein (750 µg) were taken for immunoprecipitation. The supernatants were precleared by adding Protein A/G PLUS-Agarose Immunoprecipitation Reagent (Santa Cruz Biotechnology, Inc., Dallas, Texas, USA) and incubated with IP antibody-IP matrix complexes overnight using NBS1 rabbit polyclonal antibody (Sigma Aldrich, Poznan, Poland), Mre11 rabbit monoclonal antibody (Cell Signaling, Lab-JOT Ltd., Warsaw, Poland), ATM rabbit monoclonal antibody (Epitomics, Burlingame, California, USA) according to the manufacturer’ protocol (Santa Cruz Biotechnology, Inc., Dallas, Texas, USA). Beads were washed with PBS and immune complexes were eluted with sodium dodecyl sulphate (SDS)-containing buffer and boiled. Mre11, BRCA1, ATM and NBS1 were detected using the Western blotting technique with the following antibodies: BRCA1 mouse monoclonal (R&D, Biokom, Warsaw, Poland), ATM rabbit monoclonal antibody (Epitomics, Burlingame, California, USA), Mre11 rabbit monoclonal antibody (Cell Signaling, Lab-JOT Ltd., Warsaw, Poland), NBS1 rabbit polyclonal antibody (Sigma Aldrich, Poznan, Poland) and secondary rabbit polyclonal antibody conjugated with horseradish peroxidase (Dako, Poland).

### 4. Western blotting analysis

Whole cell protein extracts were prepared according to the Laemmli method [13]. Equal amounts of protein were separated electrophorectically in 8, 12 or 15% SDS-polyacrylamide gels and afterwards transferred to nitrocellulose membranes. Membranes were blocked in 5% non-fat milk dissolved in TBS containing 0,1% Tween-20 (Sigma Aldrich, Poznan, Poland) for 1 h at RT and incubated with one of the primary monoclonal or polyclonal antibodies: anti-ATM (1∶500) (Millipore, Merck, Warsaw, Poland), anti-p-ATM Ser 1981 (1∶1000), H2AX (1∶500) and anti-γH2AX (1∶1000) (Abcam, Cambridge, UK), anti-p16 (1∶500), anti-p53 (DO-1) (1∶500), anti-p21 (C-19) (1∶500) (Santa Cruz Biotechnology Inc., Dallas, Texas, USA), anti-p-p53 Ser 15, anti-Chk1, anti-p-Chk1 Ser 317, anti-Chk2, anti-p-Chk2 Thr 68, anti-NBS1 Ser 343 (Cell Signaling, Lab-JOT Ltd., Warsaw, Poland), anti-PARP1 (1∶1000) (Becton Dickinson, Diag-med, Warsaw, Poland) anti-NBS1 (1∶500), anti-β-actin (1∶50000) (Sigma Aldrich, Poznan, Poland) and anti-GAPDH (1∶50000) (Millipore, Merck, Warsaw, Poland). The proteins were detected with appropriate secondary antibodies conjugated with horseradish peroxidase and ECL reagents (GE Healthcare, Buckinghamshire, UK), according to the manufacturer’s protocol.

### 5. Gamma irradiation procedure

Asynchronously growing cells were treated with 4 Gy of γ-irradiation. Immediately after irradiation the cells were diluted to a concentration 0,25×10^6^ cells/ml and cultured for 3 h. Cells were collected after 3 h (untreated and treated with 4 Gy of irradiation). Whole cell extracts were prepared for Western blotting analysis.

### 6. Silencing of the *NBN* gene

To downregulate *NBN* expression the cells were seeded in 6 or 12-well plates (2×10^4^ or 8×10^3^ cells per well, respectively) and transfected with 60 nM siRNA (*NBN* or negative) (Life Technologies, Warsaw, Poland) using Lipofectamine 2000 (Life Technologies, Warsaw, Poland). Transfection was performed according to the manufacturer’s protocol. About 20 h after transfection medium was replaced with fresh one and cells were cultured for three days in the presence of doxorubicin (100 nM) (Sigma Aldrich, Poznan, Poland).

### 7. Detection of SA-β-Gal

Detection of SA-β-Gal was performed according to Dimri et al. (1995) [14]. Briefly, cells were fixed with 2% formaldehyde, 0,2% glutaraldehyde in PBS, washed and exposed overnight at 37°C to a solution containing: 1 mg/ml 5-bromo-4-chloro-3-indolyl-b-D-galactopyranoside, 5 mM potassium ferrocyanide, 5 mM potassium ferrycyanide, 150 mM NaCl, 2 mM MgCl_2_ and 0,1 M phosphate buffer, pH 6,0. All of the used agents were purchased at Sigma Aldrich (Poznan, Poland). Photos were taken using the Evolutions VF digital CCD camera (Media Cybernetics, Rockville, Maryland, USA).

### 8. Apoptosis detection

The level of apoptosis was measured by flow cytometry (FACSCalibur) using the annexin V/7-AAD assay (Becton Dickinson, Diag-med, Warsaw, Poland). Externalization of phosphatidylserine (PS) to the outer layer of the cell membrane was examined by binding of annexin V in the presence of 7-AAD, a dye which stains dead cells. Briefly, cells were washed with PBS, suspended in the annexin V binding buffer, stained for 15 min with annexin V conjugated with PE and 7-AAD. Analysis was performed with FACSCalibur using the CellQuest Software (BD Biosciences, Warsaw, Poland). 10000 events were collected per sample.

### 9. Bromodeoxyuridine labeling assay

To evaluate DNA synthesis BrdU (Sigma Aldrich, Poznan, Poland) was added to the medium (10 µM) and cells were cultured for 24 h. Afterwards the cells were fixed in ethanol. BrdU was detected using a primary antibody against BrdU (Becton Dickinson, Warsaw, Poland) and a secondary Alexa 488 antibody (Life Technology, Warsaw, Poland). The cells were observed under a fluorescence microscope (Nikon, Tokyo, Japan) with the use of 450–490 nm -excitation wavelength. Photos were taken using the Evolutions VF digital CCD camera (Media Cybernetics, Rockville, Maryland, USA).

### 10. Immunocytochemistry

For immunofluorescence the cells were fixed with 2% paraformaldehyde (Sigma Aldrich, Poznan, Poland) at RT for 20 minutes and afterwards were incubated on slides with the anti-53BP1 monoclonal antibody (Novus, Cambridge, USA). Secondary anti-rabbit Alexa 488-conjugated IgG antibody was used (Life Technology, Warsaw, Poland). Cells were observed under a fluorescence microscope (Nikon, Tokyo, Japan) and photos were taken using the Evolutions VF digital CCD camera (Media Cybernetics, Rockville, Maryland, USA).

### 11. Fluorimetric Detection of DNA unwinding (FADU) method

A modified and automated version of the FADU (Fluorimetric Detection of alkaline DNA Unwinding) method was used to measure the percentage of double-stranded DNA after treatment with doxorubicin (1 and 10 µM). The percentage of DNA damage was analyzed 30, 60 and 90 min after treatment with a DNA damaging agent as described previously by Moreno-Villanueva et al. [15], [16]. The method is based on partial denaturation “unwinding” of double-stranded DNA under controlled alkaline and temperature conditions. DNA strand breaks are sites where DNA unwinding can start. Briefly, after infliction of DNA damage cell lysis was performed. DNA unwinding was terminated by adding a neutralization solution. SybrGreen, a commercially available dye, which only binds to double stranded DNA, was used to determine the amount of double-stranded DNA. The lower the fluorescence the less double-stranded DNA in the sample.

### 12. Statistical analysis

Student’s T test was used to calculate statistical significance: *, p<0,05; **, p<0,01; ***, p<0,001.

## Results

### 1. FADU analysis of L5, S3R and S4 cells treated with doxorubicin

**Figure 1:**
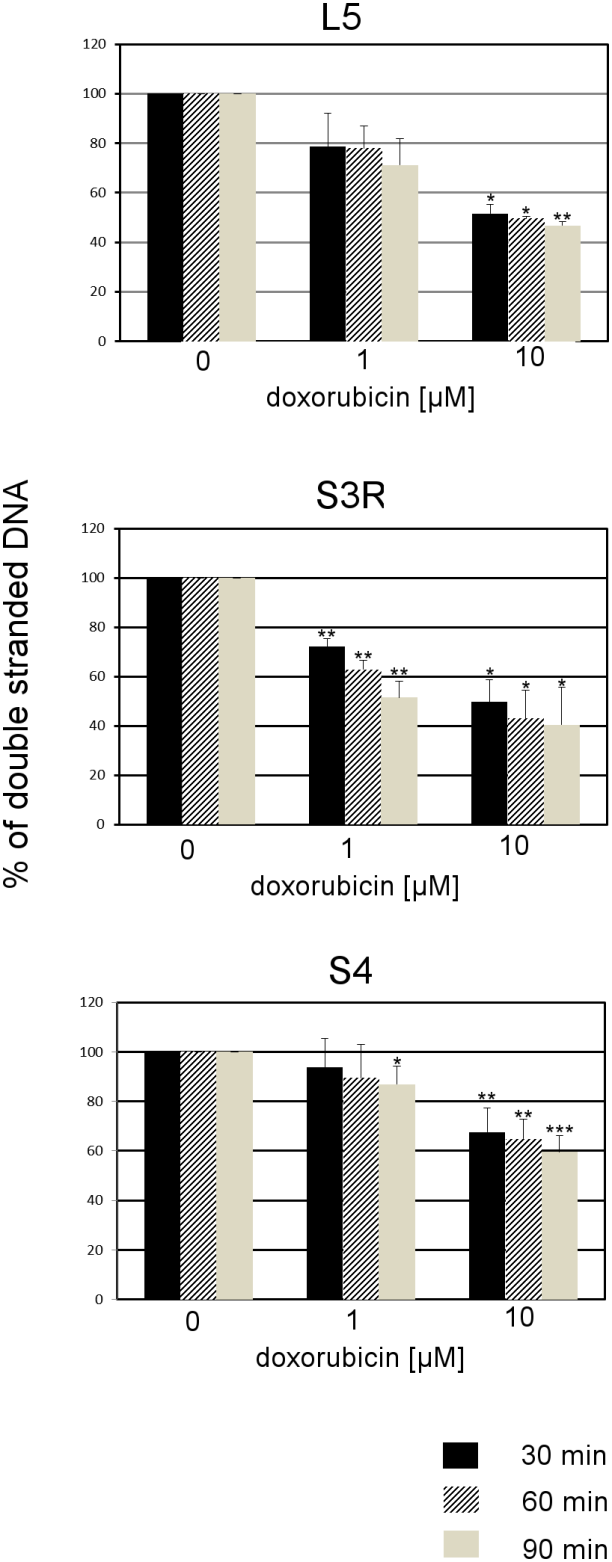
FADU analysis in L5, S3R and S4 cells treated with doxorubicin. The percentage of double-stranded DNA, was measured in all of the cell lines using the FADU method. All of the cell lines were treated with 1 and 10 µM doxorubicin and the measurements were performed 30, 60 and 90 min after treatment with this agent. Data is calculated as the percentage of control. The values are means ± SD obtained from three independent experiments. Statistical significance was estimated using the Student’s T test.

Doxorubicin is a DNA-damaging agent which is widely used in chemotheraphy. It has been shown that cytostatic doses of doxorubicin can lead to the induction of cellular senescence. Cells sensitivity to this agent can vary between different types of cells. Therefore the first step was to analyze the cells sensitivity to treatment with this agent. To do this we used the FADU method. FADU enables to measure, in an automatic way, the percentage of double-stranded DNA [15], [16], which accounts for 100% in control cells (Fig. 1). SybrGreen, a fluorescent dye used in this method, binds only to double-stranded DNA. Therefore, the less intensive fluorescence the less double-stranded DNA can be observed. To this end we treated all of the cell lines: with the mutated form of nibrin (S3R and S4) and spontaneously immortalized cells from a healthy donor (L5) with two concentrations of doxorubicin (1 and 10 µM) and analyzed the percentage of double-stranded DNA, after short periods of time (30, 60 and 90 min). The most sensitive, to treatment with doxorubicin, were the S3R cells. In the case of this cell line, a significantly lower amount of double-stranded DNA could be found at all of the analyzed time points after treatment with both concentrations of doxorubicin, in comparison with the untreated cells. In the case of S4 cell line a statistically significant decrease of the percentage of double-stranded DNA, in comparison with control cells, could be observed 90 min after treatment with the lower (1 µM) concentration of doxorubicin and in all of the time points after treatment with the higher (10 µM) concentration of this agent. This shows that even though S3R and S4 cell lines possess the same *NBN* mutation their sensitivity to treatment with doxorubicin is different. Furthermore, it turned out that, the control (L5) cells are more sensitive to doxorubicin treatment than the S4 cells, however less sensitive than the S3R cells. The obtained results allowed us to speculate that different concentrations of doxorubicin could be cytostatic for particular cell lines and different doses could be needed for the induction of doxorubicin-induced senescence.

### 2. Cell cycle arrest and apoptosis in doxorubicin-treated L5, S3R and S4 cells

**Figure 2:**
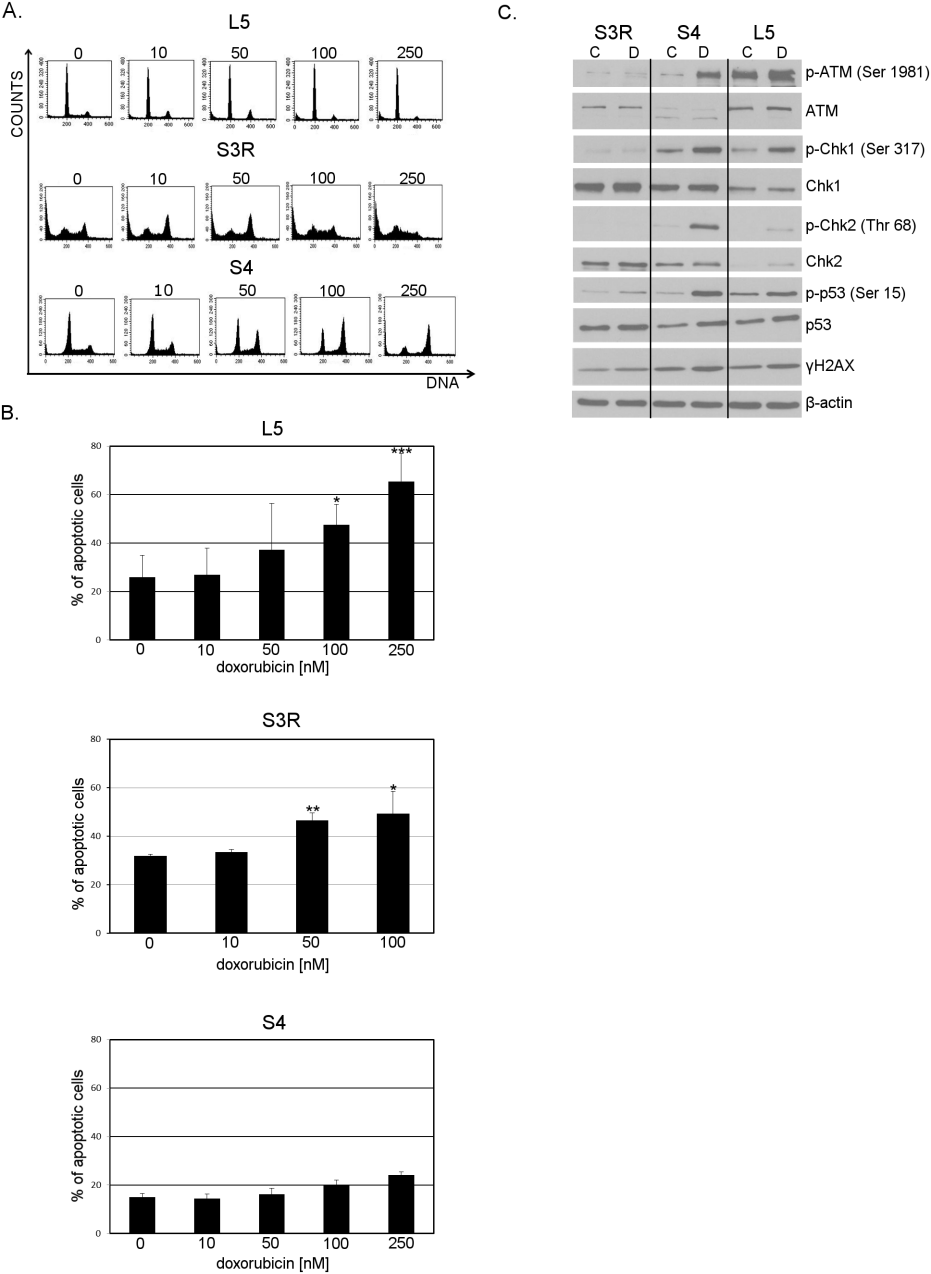
Dose-dependent influence of doxorubicin on cell cycle arrest, apoptosis and activation of the DNA damage response (DDR). **A.** DNA content, analyzed by flow cytometry, 24 h after treatment with different concentrations of doxorubicin (0–250 nM). Representative histograms from one of three independent experiments. **B.** Concentration-dependent apoptosis measured 24 h after treatment with doxorubicin (0–250 nM). The percentage of apoptotic cells was estimated by the Annexin V/7-AAD flow cytometry assay in three independent experiments. The bars show means ± SD values. Data was analyzed using the CellQuest software. Statistical significance was estimated using the Student’s T test. **C.** Expression of the DDR proteins analyzed by Western blotting in control (C) and treated with doxorubicin (D) S3R, S4 and L5 cells. Whole cell extracts were prepared 24 h after cell treatment with the following cytostatic concentrations of dox: 50 nM (L5), 10 nM (S3R), 100 nM (S4) and β-actin was used as a loading control.

One of the hallmarks of senescence is cell cycle arrest. Cells undergoing senescence can be arrested in the G1/S or G2/M phases of the cell cycle, however stress-induced premature senescence (SIPS) is predominantly associated with cell cycle arrest in the G2/M phase of the cell cycle. We treated L5, S3R and S4 cells with various concentrations of doxorubicin, ranging from 10 to 250 nM and analyzed DNA content using flow cytometry. As it is shown in Figure 2A and in Table 1 treatment with doxorubicin arrested cells from all of the cell lines in the G2/M phase of the cell cycle. In case of the control (L5) cell line the majority of cells were arrested after treatment with 50 nM doxorubicin (approximately 30%). The largest fraction of S4 cells (almost 50%) arrested in the G2/M, was observed after treatment with 100 nM doxorubicin. In the S3R cell population the majority of cells were found in the G2/M phase of the cell cycle after treatment with 10 and 50 nM doxorubicin (about 35%). The subG1 fraction which represents apoptotic cells did not exceed 11% in the case of the L5 cell line and 12% in the case of S4 cells. S3R cells were much more prone to spontaneous apoptosis and about 30% of the cells were found in the subG1 fraction. A concentration dependent increase in the level of apoptosis could be observed after treatment with doxorubicin in all of the cell lines. The DNA content analysis can underestimate the level of apoptosis due to the fact that cells with 4C DNA undergoing apoptosis, may have ≥2C DNA and cannot be distinguished from the cells found in the S and G1 phases of the cell cycle. Therefore, to estimate more accurately the percentage of cells undergoing apoptosis, we performed the Annexin V/7-AAD cytometric analysis. As expected this method revealed more apoptotic cells in all of the analyzed cell lines in comparison with the DNA content analysis. However, in the S4 cells, concentration dependence after treatment with doxorubicin still could not be observed. In the case of S3R cells about half of the cell population underwent cell death after treatment with 50 and 100 nM doxorubicin, i.e. significantly more than control cells (Fig. 2B). These results show that S3R cells are very prone to both spontaneous and doxorubicin induced apoptosis and generally more sensitive to the treatment than the S4 cells. Nonetheless, a substantial fraction of cells from all of the cell lines can be arrested in the G2/M phase of the cell cycle upon treatment with different concentrations of doxorubicin.

**Table 1:** DNA content (%) in L5, S3R and S4 cells treated with different concentration of doxorubicin.

L5						
%	untreated	10 nM	50 nM	100 nM	250 nM	
SubG1	11,47±5,32	7,18±5,85	**13,83±5,45**	20,48±5,42	24,64±9,37	
G1	59,57±5,61	65,19±3,24	**49,67±4,6**	57,49±10,15	59,28±5,42	
S	7,49±4,86	6,27±3,92	**3,92±0,21**	2,92±0,92	3,33±0,24	
G2/M	19,52±3,46	21,73±5,98	**30,1±11,14**	17,95±6,36	11,73±2,99	
**S3R**						
**%**	**untreated**	**10** **nM**	**50** **nM**	**100** **nM**	**250** **nM**	
SubG1	27,93±4,14	**29,23±5,9**	31,64±5,49	33,63±5,23	32,64±1,28	
G1	25,83±4,1	**19,04±3,9**	17,18±4,01	19,77±4,56	25,87±2,45	
S	15,57±3,72	**12,53±1,12**	10,7±1,22	15,28±1,12	16,01±0,59	
G2/M	25,08±2,09	**34,84±4,49**	35,24±4,4	26,07±3,57	19±5,57	
**S4**						
	**untreated**	**10** **nM**	**50** **nM**	**100** **nM**	**250** **nM**	**500** **nM**
SubG1	6,43±4,87	7,59±4,07	6,56±2,74	**6,72±1,57**	11,37±3,39	10,67±0,73
G1	42,01±15,9	40,37±12,17	31,63±9,31	**20,07±8,12**	18,15±2,18	35,53±1,49
S	17,13±5,69	16,67±5,85	14,12±5,28	**11±4,73**	7,68±3,56	20,78±4,08
G2/M	20,8±6,52	24,11±6,18	35,54±5,18	**48,48±5,64**	39,79±9,42	31,02±5,35

The cells were treated with doxorubicin for 24 h and DNA content was measured by flow cytometry. The percentage of DNA in all phases (including subG1) of the cell cycle is shown. Data obtained after treatment with the selected concentrations of doxorubicin, chosen for the induction of senescence, is shown in bold.

Since DNA-damage induced senescence is associated with persistant activation of the DNA-damage response (DDR) pathway, which can be observed 24–48 h after treating the cells with a DNA damage inducing agent, we decided to analyze the activation of this pathway after treating the cells with the selected concentrations of doxorubicin. Such concentrations of doxorubicin were selected which led to the accumulation of the most cells in the G2/M phase of the cell cycle and relatively low level of cell death. S3R cells were treated with 10 nM and S4 cells with 100 nM doxorubicin. For comparison we used spontaneously immortalized spleenocytes obtained from a healthy donor (L5), treated with 50 nM doxorubicin (Fig. 2C). In the case of the L5 cell line, we observed the presence of p-ATM (Ser 1981), p-p53 (Ser 15) and p-Chk1 (Ser 317), even in untreated cells. After treatment with doxorubicn (24 h) increased levels of these proteins and the presence of Chk2 and p-Chk2 (Thr 68) was observed proving the presence of an active DDR. In untreated S4 cells the phosphorylated form of Chk1 (Chk1 Ser 317) was detected. Interestingly, after treatment with doxorubicin we noticed a significant increase in the level of p-ATM (Ser 1981), p-Chk1 (Ser 317), p-Chk2 (Thr 68), p-p53 (Ser 15) and γH2AX. Our results show that upon treatment with doxorubicin the DDR pathway is only activated in the L5 and S4 cell lines, however this process can’t be observed in the S3R cell line.

### 3. Doxorubicin-induced senescence of L5 and S4, but not S3R cells

**Figure 3:**
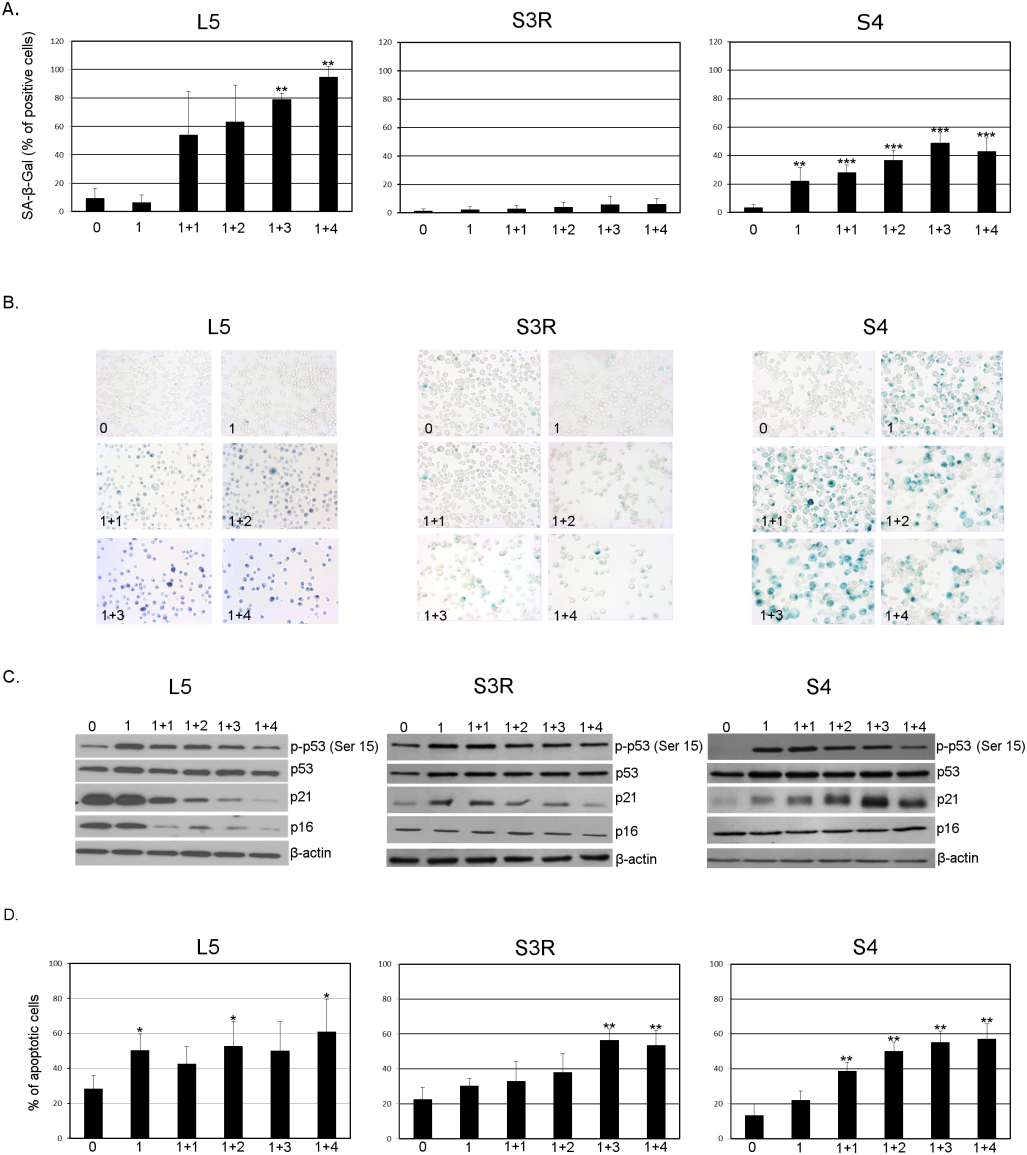
The induction of cellular senescence and apoptosis upon doxorubicin treatment. **A.** SA-β-Gal activity. Cells were treated for 1 day with doxorubicin (L5 - 50 nM, S3R - 10 nM and S4 - 100 nM) and then cultured in doxorubicin-free medium (1+n; n- are days of culture without doxorubicin). The bars show means ± SD. The percentage of SA-β-Gal positive cells from at least three independent experiments and representative images from one of three independent experiments. **B.** Magnification 200x. **C.** Expression of protein markers of senescence (p-p53, p53, p21 and p16) analyzed by Western blotting in untreated (0) cells and on the following days after culturing in doxorubicin-free medium (1+n), β-actin was used as a loading control. **D.** The percentage of apoptotic cells after treatment with doxorubicin estimated by the AnnexinV/7-AAD flow cytometry assay. The bars show means ± SD values. Data were obtained from three independent experiments. Statistical significance was estimated using the Student’s T test.

There is data showing that immortalized and cancer cells retain the ability to undergo senescence including that induced by DNA damage [17], [18]. We have previously shown [19] that treatment of human colon cancer HCT116 cells with a low dose of doxorubicin for one day, followed by culture in a drug-free medium, led to the induction of senescence. Therefore, we decided to use the same experimental approach and treated the L5, S3R and S4 cells with the chosen concentrations of doxorubicin (50 nM for L5, 10 nM for S3R and 100 nM for S4) for 24 hours and afterwards cultured the cells for four days in a drug free medium (1+4). We observed a time-dependent increase in the number of SA-β-Gal positive cells in L5 and S4, but not in S3R cell line (Fig. 3A, B). In case of the L5 cell line the majority of SA-β-Gal-positive cells (approximately 95%) were observed on day 1+4. In case of the S4 cell line the most SA-β-Gal positive cells were observed on day 1+3 (approximately 50%). The presence of SA-β-Gal positive cells was accompanied by an increase in the level of p53 (Ser 15) in both cell lines, however a time dependent increase in the level of p21 was only observed in the S4 cell line. Surprisingly, in the L5 cell line, a time dependent decrease in the level of this protein was found (Fig. 3C). Two crucial pathways play an important role in senescence: p53-p21 and p16-pRb. Sometimes these pathways overlap therefore we also decided to check the level of p16, which is a key protein in the p16-pRb pathway.

The p16-mediated senescence acts mainly through the retinoblastoma (pRb) pathway by inhibiting the action of the cyclin dependent kinases and leads to G1 cell cycle arrest [20]. We did not observe any changes in the level of this protein in the S3R and S4 cell lines, however, a time dependent decrease in the level of p16 was observed in the L5 cell line (Fig. 3C). The observation made in the L5 cell line requires further elucidation. It seems that untreated S3R cells might have the p53/p21 pathway already active, which is further slightly activated after treatment with doxorubicin, but this is not accompanied by an increase in SA-β-Gal activity. In the L5 and S4 cells stronger activation of the p53/p21 pathway correlated with an increase in SA-β-Gal activity. This encouraged us to investigate whether the lack of induction of senescence in the S3R cell line was due to the fact that the cells underwent cell death. Using the annexin V/7-AAD assay, we measured the level of apoptosis a day after treatment with doxorubicin and on subsequent days after transferring the cells to fresh medium (Fig. 3D). In all of the cell lines we observed a time-dependent increase in the level of apoptosis. Three days after culturing the cells in drug free medium (1+3) approximately 55% of cells underwent apoptosis in all of the cell lines, however it should be underlined that the cells were treated with different concentrations of doxorubicin. The S4 cells were treated with a ten times higher concentration of doxorubicin (100 nM) than the S3R cells (10 nM). Moreover, in the case of the L5 and S3R cell lines a high basal level of apoptosis could be observed. Despite the high level of cell death, a fraction of S4 (more than 40% of SA-β-Gal positive cells on day 1+4) and L5 (about 95% of SA-β-Gal positive cells on day 1+4) of cells, that survived, were able to undergo senescence.

### 4. The level of p70-nibrin in S3R and S4 cells

**Figure 4:**
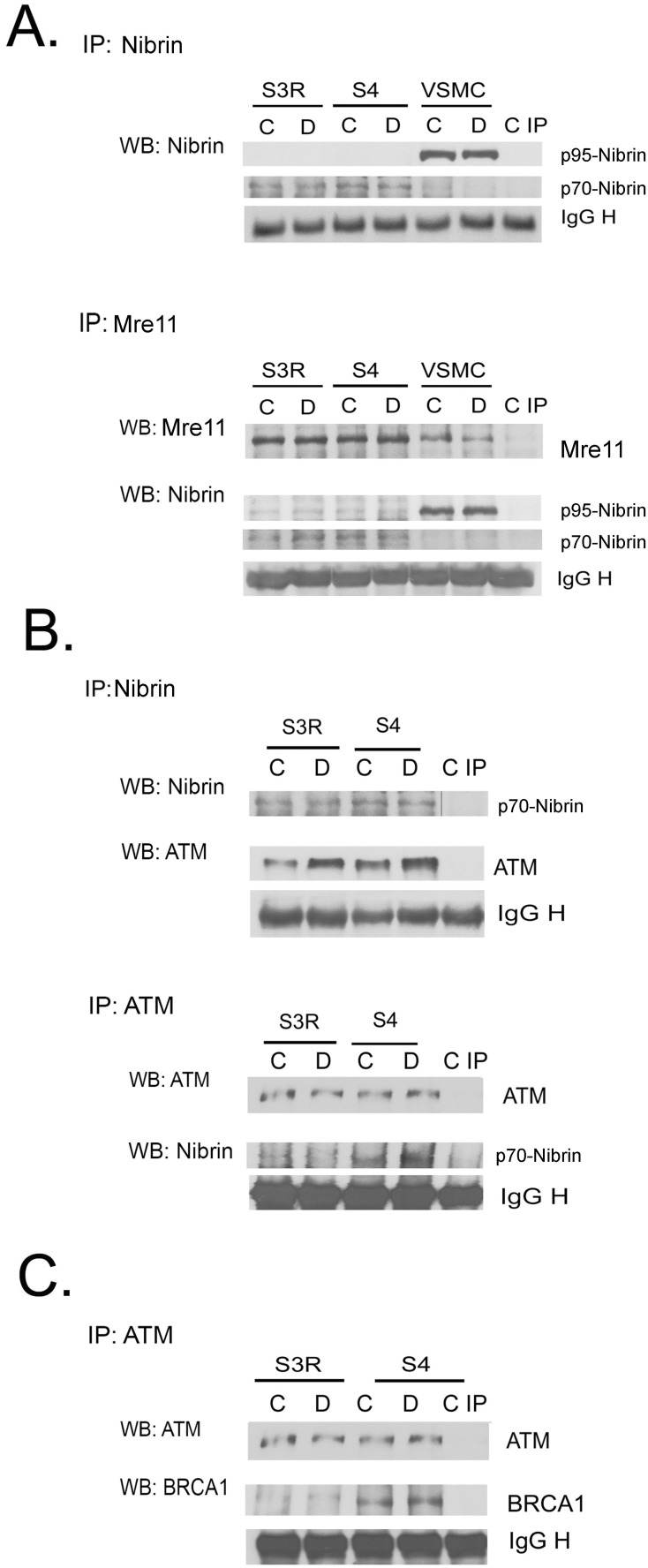
Levels of nibrin, p70-nibrin, MRE11, ATM and BRCA1 in the DDR complex estimated by immunoprecipitation assay. **A.** Level of nibrin: wild-type (p95) and the truncated form (p70) in control (C) and doxorubicin treated (D, 1 µM/1 h) S3R, S4 and VSMCs. Expression of nibrin was analyzed by immunoprecipitation using an anti-NBS1 antibody followed by Western blotting with anti-NBS1 (upper panel). Alternatively, IP using anti-MRE11 antibody was performed followed by WB with anti-NBS1 (lower panel). MRE11 was used as a loading control. The last lane (C IP) shows the negative IP control. Note that p95 is only present in VSMCs, in which there is no p70-nibrin. **B.** ATM binding to nibrin in control and doxorubicin-treated S3R and S4 cells analyzed by immunoprecipitation using anti-NBS1 antibody (upper panel) or anti-ATM antibody (lower panel). Levels of ATM and p70 were detected by WB. Loading controls were performed in both variants of IP. **C.** Expression of BRCA1 in control and dox-treated S3R and S4 cells was analyzed by immunoprecipitation using anti-ATM antibody followed by WB using an anti-BRCA1 antibody. Loading and negative IP controls were performed as above.

We were interested whether the differences in the cells susceptibility to doxorubicin treatment and cell fate were due to a different level of the truncated form of nibrin (p70), which is present in the S3R and S4 cells. To elucidate this we performed an immunoprecipitation assay which showed the same level of p70-nibrin in both untreated and doxorubicin treated S3R and S4 cells (Fig. 4A). The p95 form of nibrin was not detected neither in S3R nor in S4 cells, however it was observed in VSMC cells, which were used as a positive control. To confirm the above observation and to exclude the possibility that the amount of immunoprecipitated protein was not equal, we verified the p70-nibrin level also after IP, using the anti-Mre11 antibody. In this case we checked the level of Mre11 by WB, as a loading control, and followed with analysis of nibrin. Also this time we did not detect any differences in the level of p70-nibrin. To verify the functionality of the truncated nibrin we analyzed its binding to ATM. After immunoprecipitating either ATM or nibrin it was observed that p70-nibrin was able to form a complex with ATM in both NBS1 deficient cell lines (Fig. 4B). However, the IP revealed that more ATM immunoprecipitated with p70-nibrin in S4 than S3R cells. This may suggest that formation of DNA damage-induced ATM-nibrin complex is more efficient in S4 cells. This difference was already found in untreated cells and correlated with the observed higher phosphorylation of ATM in response to doxorubicin treatment of S4 cells (Fig. 2C). The possible better function of the DNA damage/repair response in S4 cells was confirmed by a further IP experiment showing that in these cells more BRCA1 was immunoprecipitated with ATM (Fig. 4C) suggesting that S4 are more efficient in DNA repair than the S3R cells.

### 5. Radiation induced activation of the DDR pathway in L5, S3R and S4 cells

**Figure 5:**
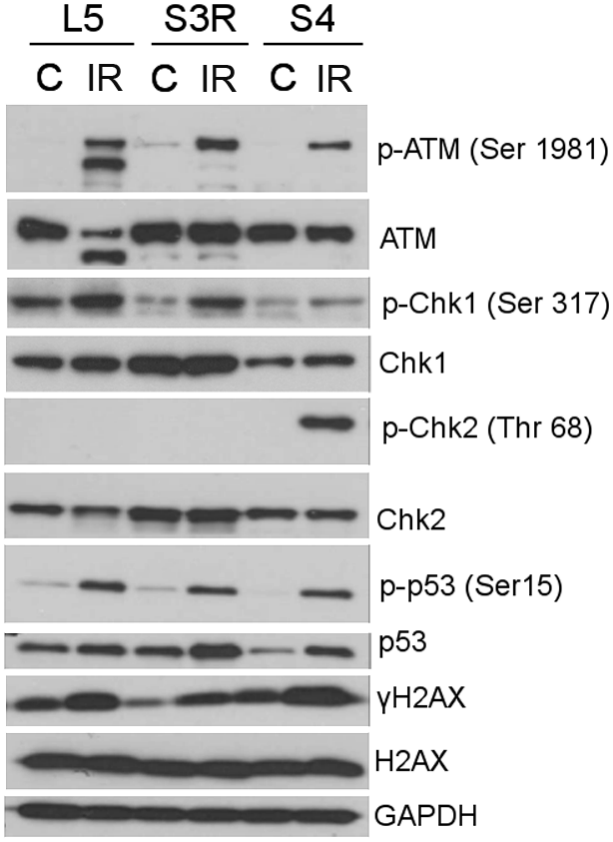
Activation of the DNA damage response pathway upon γ-irradiation. Expression of the DDR proteins analyzed by Western blotting in control (C) and exposed to radiation (IR) S3R, S4 and L5 cells. Whole cell extracts were prepared 3 h after exposure to 4 Gy of γ-radiation, β-actin was used as a loading control.

Despite the fact that Nijmegen Breakage Syndrome was caused by the same mutation in the S3R and S4 cell lines their susceptibility to doxorubicin treatment differed. To verify whether this was a characteristic feature of only doxorubicin, we used a different DNA-damaging agent. Therefore the cells ability to activate the DDR pathway after being exposed to γ-radiation (4Gy and cultured for 3 h) was analyzed (Fig. 5). Interestingly, exposure to γ-radiation of both S4 and S3R as well as control (L5) cells led to an efficient induction of DDR. An increase in the level of the following proteins was observed in all of the analyzed cell lines: p-ATM (Ser 1981), p-Chk1 (Ser 317), p-p53 (Ser 15) and γH2AX. The phosphorylated form of Chk2 (Thr 68) was only noticed upon exposure to γ-radiation of the S4 cells. This indicated that these cells retain the capacity to upregulate the components of the DDR pathway, at least for a short period of time.

### 6. The role of nibrin in DNA damage induced senescence of human Vascular Smooth Muscle Cells

**Figure 6:**
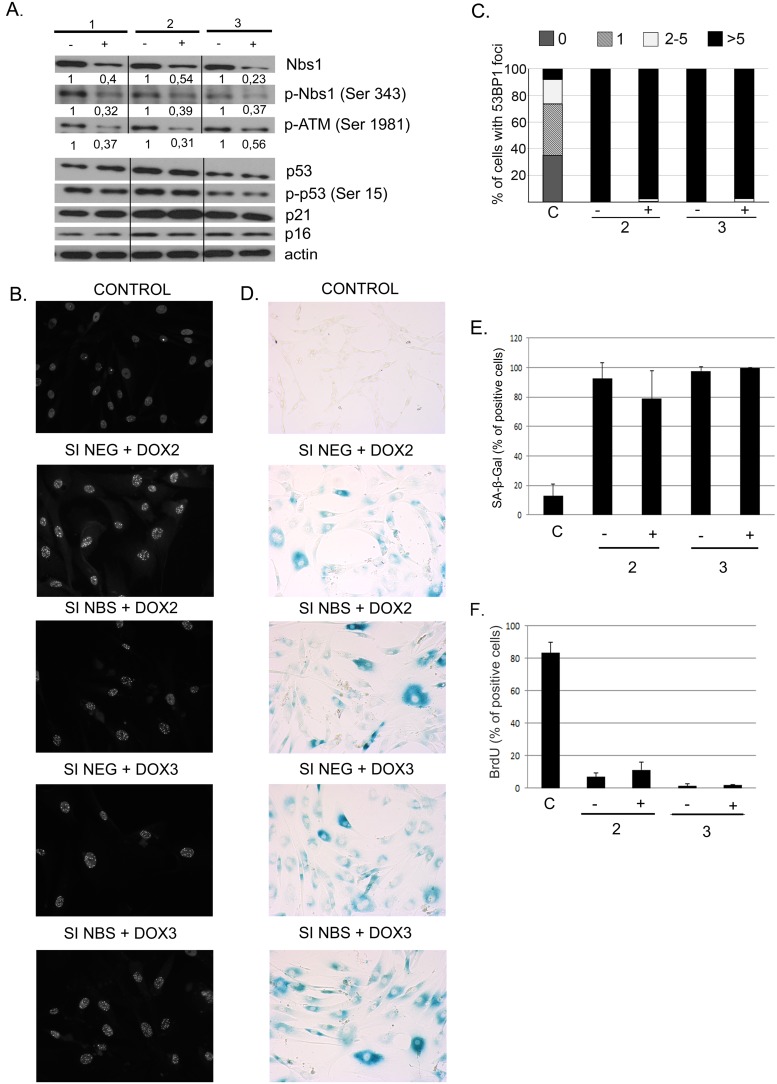
The role of nibrin in doxorubicin-induced senescence of human Vascular Smooth Muscle Cells (VSMCs). Cells were transfected with negative siRNA (−) or *NBN* siRNA (+) and afterwards cultured for three days in the presence of doxorubicin (100 nM). **A.** Downregulation of the NBS1 protein level in VSMCs using specific siRNA (60 nM). Whole cell extracts were prepared at indicated time points after treatment with doxorubicin. Expression of the indicated proteins was estimated by Western blotting, β-actin was used as a loading control. The amount of the protein in cells transfected with *NBN* siRNA was calculated by densitometry as a fraction of that present in cells transfected with negative siRNA (1). **B.** 53BP1 staining in doxorubicin-treated control cells and cells with silenced nibrin. Representative images from one of three independent experiments. Magnification 100x. **C.** 53BP1 staining in doxorubicin treated control cells and cells with silenced nibrin. Cells with DNA damage were divided into four groups based on the number of 53BP1 foci: cells without 53BP1 foci, with one focus, with 2–5 foci, with more than 5 foci. Means from three independent experiments. **D.** SA-β-Gal activity in doxorubicin treated VSMC cells. Representative images from one of three independent experiments, magnification 100x. **E.** The percentage of SA-β-Gal positive cells (a mean ± SD) from three independent experiments. **F.** BrdU incorporation assay. Control cells and cells transfected with negative siRNA or *NBN* si RNA were cultured with BrdU for 24 h. Data presented as means ± SD from three independent experiments.

Since a relatively low level of the truncated nibrin (p70-nibrin) in S4 cells was sufficient for activation of the DDR signaling pathway followed by senescence, we asked whether downregulation of the NBS1 protein in normal cells would influence DDR activation and senescence upon treatment with doxorubicin. Transfection of L5 cells, using the nucleofection method, turned out to be unsuccessful. Only 25% of the transfected cells were viable 24 h after transfection. Therefore to analyze the effect of downregulation of nibrin on the induction of senescence, we used vascular smooth muscle cells (VSMCs) which were shown by us to undergo senescence after treatment with doxorubicin [21]. Before treatment with doxorubicin, the cells were transfected with negative siRNA or *NBN* siRNA with 85% transfection efficiency measured a day after transfection (not shown). As shown in Figure 6A the level of NBS1 in cells transfected with *NBN* siRNA and cultured in the presence of doxorubicin for three days was reduced from two to four times. Moreover the levels of p-NBS1 (Ser 343) and p-ATM (Ser 1981) were substantially reduced in these cells. However, there were no differences in the level of p53 and p21 proteins between cells transfected with negative siRNA and *NBN* siRNA (Fig. 6A). Next we decided to verify whether the downregulation of nibrin would affect the formation of 53BP1 foci, after treatment with doxorubicin. Recently 53BP1 has been recognized as a convenient marker of DSBs [22]. We observed that the formation of 53BP1 foci was not affected when the level of NBS1 was reduced (Fig. 6B, 6C). This could suggest that senescence was also not affected in cells with reduced level of NBS1. Indeed, the percentage of SA-β-Gal positive cells was substantially increased already two days after treatment with doxorubicin in both types of cells and accounted for 100% on day 3 of treatment with doxorubicin (Fig. 6D, 6E). These results were confirmed using the BrdU incorporation assay, which showed complete inhibition of proliferation in cells which were transfected with negative siRNA and *NBN* siRNA and subsequently treated with doxorubicin (Fig. 6F).

Taken together, the obtained results performed on human VSMCs indicate that a substantially reduced level of NBS1 did not influence doxorubicin-induced DDR and senescence in these cells.

## Discussion

The aim of our study was to investigate the role of nibrin in doxorubicininduced senescence.

Cellular senescence is associated withpermanent growth arrest. We can distinguish two types of cellular senescence: replicative which is telomere shortening dependent and stress-induced premature senescence, which is telomere shortening independent. Replicatively senescing cells are believed to activate the G1 restriction point. However, it was recently documented that replicative senescence can stop the cells in both the G1/S and G2/M phases of the cell cycle [23] while SIPS is mainly associated with cell cycle arrest cells in the G2/M phase of the cell cycle.

NBS1 deficient cells have improperly functioning cell cycle checkpoints [24], including a defect of the DNA damage induced intra-S-phase checkpoint which is responsible for the radio-resistant DNA synthesis (RDS)- a continuation of DNA synthesis despite the presence of radiation-induced DNA damage [25]. However, the reports concerning the status of cell cycle checkpoints in NBS deficient cells are discrepant, since both impaired and normal G1/S or G2/M arrest after cell irradiation have been reported (reviewed by [26]). Previously it was documented that S3R cells had a reduced capacity to undergo G1 arrest and showed a marked accumulation of cells in the G2/M phase of the cell cycle 24 h after 4Gy γ-irradiation, though to a lesser extent than the S4 cells [27]. We have shown that treatment with doxorubicin of L5, S3R and S4 cells led to an arrest in the G2/M phase of the cell cycle. However, we proved that S3R cells had a less efficient G2 checkpoint than S4 cells. Treatment of S3R cells with 100 nM doxorubicin, the concentration which halted most of the S4 cells in the G2/M phase of the cell cycle, led to massive cell death of S3R cells. Nevertheless the percentage of S3R cells which were arrested in the G2/M phase of the cell cycle, after treatment with the selected, cytostatic concentrations of doxorubicin, was comparable to the one observed in control L5 cells.

The higher propensity of S3R than S4 cells to undergo apoptosis was connected with a decrease in the level of double-stranded DNA as revealed using the FADU method. One should keep in mind that doxorubicin is a DNA-damaging agent which acts through different mechanisms. Amongst all, it induces the formation of cross-links which prevent DNA from unwinding. The FADU method enables to measure DNA susceptibility to unwind, which is a function of the number of chromatin modifications. Therefore the FADU method, in the context of this particular agent, can only be used as a screening method which enables to verify the cells susceptibility to treatment with different concentrations of doxorubicin. Nevertheless, the decrease in the amount of double-stranded DNA was observed with the increasing concentrations of doxorubicin and time of treatment in all of the three examined cell lines, proving that at least a portion of DNA acquires double strand breaks upon treatment with doxorubicin. Generally, NBS1 deficient cells have impaired DNA repair. This process seems to be more severe in the S3R than in the S4 cells due to the lower level of the BRCA1 protein, which doesn’t interact with ATM in the S3R cells. It was reported that downregulation of the NBS1 protein level by siRNA led to an increase in irradiation-induced mutation frequency in human lymphoblastoid cells [26]. Moreover, it is worth to note that null mutation of *Nbs* is lethal in mice [4].

Interestingly, the presence of less double-stranded DNA, after treatment with doxorubicin, in the S3R cells, than in the S4 and L5 cells, was not linked to ATM activation. However, we observed increased levels of p-ATM and its downstream targets such as p-Chk1, p-p53 and γH2AX 24 h after treatment with doxorubicin in control (L5) and S4 cells. Moreover in the S4 cells upon doxorubicin treatment, a substantial increase in the level of p-Chk2 (Thr 68) could be seen. Surprisingly all of the cell lines retained the ability to activate DDR upon exposure to γ-radiation. Several studies showed severe impairment of the DDR activation in NBS1 deficient cells. Namely, cells from NBS patients have been reported to be deficient in ATM phosphorylation of p53, Chk2 and other substrates following DNA damage. Other studies showed that the C-terminal fragment of nibrin was sufficient to stimulate ATM activation at early times after irradiation. In contrast, nuclear expression of a nibrin transgene lacking the C-terminal 100 amino acids was unable to stimulate ATM activation under the same conditions ([28] and literature there). This was most likely due to the lack of the ATM binding domain. We have shown that despite the presence of the same *NBN* gene mutation, DDR is only activated in the S4 cells. Furthermore, this pathway was also activated in the L5 cells. In S3R cells some elements of the DDR (p-p53, p-Chk1 and p-Chk2) were already present in untreated cells and 24 h treatment with doxorubicin did not lead to an increase in the level of these proteins. It is tempting to speculate that the different response of the two NBS1 deficient cell lines to treatment with doxorubicin is caused by the presence of a lower level and/or nonfunctional truncated form of nibrin (p70-nibrin) in S3R cells. Indeed, it has been shown that the level of p70-nibrin can vary in cells obtained from NBS patients [2]. However, our results showed the same amount of p70-nibrin in S4 and S3R cells. Moreover, in both cell lines p70-nibrin co-immunoprecipitated with ATM. Nevertheless we observed that a higher level of p70-nibrin precipitated with ATM in S4 cells than in the S3R cells. In contrast to the results obtained using the S4 cells and the L5 cells with wild-type *NBN* gene, we did not observe ATM phosphorylation after treatment with doxorubicin in S3R cells. On the other hand, a low level of the phosphorylated form of p53 (p-p53 Ser 15) was detected in untreated, S3R cells and its level increased after treatment with doxorubicin. Others [29] showed impaired, but still detectable, ATM and p53 phosphorylation in doxorubicin-treated NBS fibroblasts. Interestingly, in NBS fibroblasts the p26 instead of the p70 fragment of nibrin could be found, which doesn’t possess the ATM binding domain. This discrepancy could be explained by the fact that p53 can be phosphorylated on Ser 15 not only by ATM, but also by DNA-PK, which plays a vital role in DSB repair as well as in driving cells to apoptosis [30]. Nonetheless, the results obtained by Hou et al. [29] allowed to conclude that NBS1 is acting upstream of ATM. On the other hand, ATM phosphorylates nibrin at its Ser 343 residue [7]. We showed that nibrin can act both downstream and upstream of ATM, as downregulation of nibrin affected phosphorylation of both nibrin and ATM. These results suggested that DDR could be compromised in cells with a diminished level of nibrin. However, in VSMCs, in which the level of nibrin was substantially reduced, the p53/p21 pathway was practically not affected which suggests that in normal cells there must be a redundancy of this protein. Surprisingly, despite the presence of the same amount of p70-nibrin in both cell lines, the p53/p21 pathway was only activated in the S4 cells. This could imply a failure in DDR activation downstream of ATM in the S3R cells. However, these cells had much less ATM bound to nibrin in the IP assay.

Moreover, we detected a higher basal level of apoptosis in control S3R cells, but a substantially lower level of the BRCA1 protein in comparison with S4 cells in the IP assay. This indicates that S3R cells could have a limited capacity for DNA repair what could be reflected by a very high rate of spontaneous apoptosis in these cells. Indeed, also the basal level of p-p53 was higher in S3R than in S4 cells indicating p53-dependent apoptosis.

It seems that DDR can be a culprit of cell senescence, therefore we wondered whether S3R cells would be able to senesce after treatment with doxorubicin. Indeed, in both L5 and S4 cells we observed the appearance of the common and widely used marker of senescence, which is increased SA-β-Gal activity. The presence of this marker of senescence is common in adherent cells [5] however data concerning senescence of lymphoid cells and the presence of this hallmark is scarce [31]. Additionally increased activity of SA-β-Gal in the S4 cells was accompanied by a time-dependent increase in the level of p21, which is a cdk inhibitor. Thus, we can conclude that L5 and S4 cells, contrary to S3R cells, are able to activate the DDR and undergo senescence. Moreover VSMCs with a highly reduced level of nibrin were also able to undergo senescence just like cells with the proper level of this protein. We can speculate that there is a minimal amount of nibrin or its truncated p70 form which is indispensable for the activation of DDR and the subsequent induction of senescence. Interestingly, it has been shown very recently that doxorubicin treated ATM-deficient human fibroblasts underwent Akt-dependent SIPS without DDR activation [32]. It seems that S3R cells are unable to activate such a program and, most likely any senescence pathway.

We showed that S3R cells are generally more sensitive to doxorubicin treatment than the S4 and L5 cell lines. Also others showed extreme variations in the propensity to undergo DNA damage-induced apoptosis (40-fold) amongst lymphoid cells derived from the NBS patients [33]. The authors did not find a correlation between the propensity to undergo apoptosis and the level of the truncated form of nibrin-p70. The mechanisms of cell death in these cells is still awaiting elucidation.

It seems that, despite the presence of a similar level of p70-nibrin, in the S3R and S4 cell lines, the differences in ATM phosphorylation and its ability to bind nibrin were crucial for the efficient activation of DDR and the induction of senescence. We observed that some proteins which are involved in the DNA damage/repair pathway (ATM, BRCA1) were more efficiently recruited to the DNA damage-induced complex in S4 than in S3R cells what might explain the differences in the cell fate after treatment with doxorubicin.

Moreover it cannot be excluded that the described in this paper differences in the S3R and S4 cell phenotype, may result from genomic instability of patients with Nijmegen Breakage Syndrome or the immortalization process. It has also been previously shown that NBS patients with the same genotype may vary in the phenotypic expression [34]. It is worth to note that unsupervised clustering of whole genome gene expression arrays of S3R and S4 cells indicated that common gene expression changes, between the two lines, also exist [35].

## References

[1] Chrzanowska KH, Gregorek H, Dembowska-Baginska B, Kalina MA, Digweed M (2012) Nijmegen breakage syndrome (NBS). Orphanet J Rare Dis 7: 1–19. 22373003 10.1186/1750-1172-7-13 PMC3314554

[2] Kruger L, Demuth I, Neitzel H, Varon R, Sperling K, et al (2007) Cancer incidence in Nijmegen breakage syndrome is modulated by the amount of a variant NBS protein. Carcinogenesis 28: 107–111. 16840438 10.1093/carcin/bgl126

[3] Maser RS, Wong KK, Sahin E, Xia H, Naylor M, et al (2007) DNA-dependent protein kinase catalytic subunit is not required for dysfunctional telomere fusion and checkpoint response in the telomerase-deficient mouse. Mol Cell Biol 27: 2253–2265. 17145779 10.1128/MCB.01354-06 PMC1820500

[4] Zhu J, Petersen S, Tessarollo L, Nussenzweig A (2001) Targeted disruption of the Nijmegen breakage syndrome gene NBS1 leads to early embryonic lethality in mice. Curr Biol 11: 105–109. 11231126 10.1016/s0960-9822(01)00019-7

[5] Sikora E, Arendt T, Bennett M, Narita M (2011) Impact of cellular senescence signature on ageing research. Ageing Res Rev 10: 146–152. 20946972 10.1016/j.arr.2010.10.002

[6] d’Adda di Fagagna F (2008) Living on a break: cellular senescence as a DNA-damage response. Nat Rev Cancer 8: 512–522. 18574463 10.1038/nrc2440

[7] Kobayashi J, Tauchi H, Sakamoto S, Nakamura A, Morishima K, et al (2002) NBS1 localizes to gamma-H2AX foci through interaction with the FHA/BRCT domain. Curr Biol 12: 1846–1851. 12419185 10.1016/s0960-9822(02)01259-9

[8] Freedman DA (2005) Senescence and its bypass in the vascular endothelium. Front Biosci 10: 940–950. 15569632 10.2741/1588

[9] Siwicki JK, Degerman S, Chrzanowska KH, Roos G (2003) Telomere maintenance and cell cycle regulation in spontaneously immortalized T-cell lines from Nijmegen breakage syndrome patients. Exp Cell Res 287: 178–189. 12799193 10.1016/s0014-4827(03)00140-x

[10] Gewirtz DA (1999) A critical evaluation of the mechanisms of action proposed for the antitumor effects of the anthracycline antibiotics adriamycin and daunorubicin. Biochem Pharmacol 57: 727–741. 10075079 10.1016/s0006-2952(98)00307-4

[11] Siwicki JK, Hedberg Y, Nowak R, Loden M, Zhao J, et al (2000) Long-term cultured IL-2-dependent T cell lines demonstrate p16(INK4a) overexpression, normal pRb/p53, and upregulation of cyclins E or D2. Exp Gerontol 35: 375–388. 10832057 10.1016/s0531-5565(00)00088-7

[12] Keeshan K, Mills K, Cotter TG, McKenna SL (2001) Elevated Bcr-Abl expression levels are sufficient for a haematopoietic cell line to acquire a drug-resistant phenotype. Leukemia 15: 1823–1833. 11753601 10.1038/sj.leu.2402309

[13] Laemmli UK (1970) Cleavage of structural proteins during the assembly of the head of bacteriophage T4. Nature. 227: 680–685. 10.1038/227680a0 5432063

[14] Dimri GP, Lee X, Basile G, Acosta M, Scott G, et al (1995) A biomarker that identifies senescence human cells in culture and in aging skin *in* *vivo* . Proc Natl Acad Sci USA 92: 9363–9367. 7568133 10.1073/pnas.92.20.9363 PMC40985

[15] Moreno-Villanueva M, Eltze T, Dressler D, Bernhardt J, Hirsch C (2011) The automated FADU-assay, a potential high-throughput in vitro method for early screening of DNA breakage. Altex 28: 295–303. 22130482 10.14573/altex.2011.4.295

[16] Moreno-Villanueva M, Pfeiffer R, Sindlinger T, Leake A, Muller M (2009) A modified and automated version of the ‘Fluorimetric Detection of Alkaline DNA Unwinding’ method to quantify formation and repair of DNA strand breaks. BMC Biotechnol 9: 39. 19389244 10.1186/1472-6750-9-39 PMC2679009

[17] Sherman MY, Meng L, Stampfer M, Gabai VL, Yaglom JA (2011) Oncogenes induce senescence with incomplete growth arrest and supress the DNA damage response in immortalized cells. Aging Cell 10(6): 949–961. 21824272 10.1111/j.1474-9726.2011.00736.x PMC3433764

[18] Shay JW, Roninson IB (2004) Hallmarks of senescence in carcinogenesis and cancer therapy. Oncogene 23: 2919–2933. 15077154 10.1038/sj.onc.1207518

[19] Sliwinska MA, Mosieniak G, Wolanin K, Babik A, Piwocka K, et al (2009) Induction of senescence with doxorubicin leads to increased genomic instability of HCT116 cells. Mech Ageing Dev 130: 24–32. 18538372 10.1016/j.mad.2008.04.011

[20] Rayess H, Wang MB, Srivatsan ES (2012) Cellular senescence and tumor suppressor gene p16. Int J Cancer 130: 1715–1725. 22025288 10.1002/ijc.27316 PMC3288293

[21] Bielak-Zmijewska A, Wnuk M, Przybylska D, Grabowska W, Lewinska A, et al (2014) A comparison of replicative senescence and doxorubicin-induced premature senescence of vascular smooth muscle cells isolated from human aorta. Biogerontology 15: 47–64. 24243065 10.1007/s10522-013-9477-9 PMC3905196

[22] Schultz LB, Chehab NH, Malikzay A, Halazonetis TD (2000) p53 binding protein 1 (53BP1) is an early participant in the cellular response to DNA double-strand breaks. J Cell Biol 151: 1381–1390. 11134068 10.1083/jcb.151.7.1381 PMC2150674

[23] Mao Z, Ke Z, Gorbunova V, Seluanov A (2012) Replicatively senescent cells are arrested in G1 and G2 phases. Aging 4: 431–435. 22745179 10.18632/aging.100467 PMC3409679

[24] Shiloh Y (1997) Ataxia-telangiectasia and the Nijmegen breakage syndrome: related disorders but genes apart. Annu Rev Genet 31: 635–662. 9442910 10.1146/annurev.genet.31.1.635

[25] Falck J, Petrini JH, Williams BR, Lukas J, Bartek J (2002) The DNA damage-dependent intra-S phase checkpoint is regulated by parallel pathways. Nat Genet 30: 290–294. 11850621 10.1038/ng845

[26] Zhang Y, Zhou J, Lim CU (2006) The role of NBS1 in DNA double strand break repair, telomere stability, and cell cycle checkpoint control. Cell Res 16: 45–54. 16467875 10.1038/sj.cr.7310007

[27] Siwicki JK, Berglund M, Rygier J, Pienkowska-Grela B, Grygalewicz B, et al (2004) Spontaneously immortalized human T lymphocytes develop gain of chromosomal region 2p13–24 as an early and common genetic event. Genes Chromosomes Cancer 41: 133–144. 15287026 10.1002/gcc.20059

[28] Cerosaletti K, Wright J, Concannon P (2006) Active role for nibrin in the kinetics of atm activation. Mol Cell Biol 26: 1691–1699. 16478990 10.1128/MCB.26.5.1691-1699.2006 PMC1430256

[29] Hou YY, Toh MT, Wang X (2012) NBS1 deficiency promotes genome instability by affecting DNA damage signaling pathway and impairing telomere integrity. Cell Biochem Funct 30: 233–242. 22161642 10.1002/cbf.1840

[30] Hill R, Lee PW (2010) The DNA-dependent protein kinase (DNA-PK): More than just a case of making ends meet? Cell Cycle 9: 3460–3469. 20855954 10.4161/cc.9.17.13043

[31] Chebel A, Bauwens S, Gerland LM, Belleville A, Urbanowicz I, et al (2009) Telomere uncapping during in vitro T-lymphocyte senescence. Aging Cell 8: 52–64. 19077045 10.1111/j.1474-9726.2008.00448.x

[32] Park J, Jo YH, Cho CH, Choe W, Kang I, et al (2013) ATM-deficient human fibroblast cells are resistant to low levels of DNA double-strand break induced apoptosis and subsequently undergo drug-induced premature senescence. Biochem Biophys Res Commun 430: 429–435. 23178571 10.1016/j.bbrc.2012.11.040

[33] Thierfelder N, Demuth I, Burghardt N, Schmelz K, Sperling K, et al (2008) Extreme variation in apoptosis capacity amongst lymphoid cells of Nijmegen breakage syndrome patients. Eur J Cell Biol 87: 111–121. 17977616 10.1016/j.ejcb.2007.09.002

[34] Nijmegen breakage syndrome (2000) The International Nijmegen Breakage Syndrome Study Group. Arch Dis Child. 82, 400–406. 10.1136/adc.82.5.400 PMC1718318 10799436

[35] Degerman S, Siwicki JK, Osterman P, Lafferty-Whyte K, Keith WN, et al (2010) Telomerase upregulation is a postcrisis event during senescence bypass and immortalization of two Nijmegen breakage syndrome T cell cultures. Aging Cell 9: 220–235. 20089118 10.1111/j.1474-9726.2010.00550.x

